# OTC analgesics and drug interactions: clinical implications

**DOI:** 10.1186/1750-4732-2-2

**Published:** 2008-02-07

**Authors:** A Mark Fendrick, Deborah E Pan, Grace E Johnson

**Affiliations:** 1 Professor, Internal Medicine/Health Management and Policy, University of Michigan Medical Center, 300 North Ingalls Building, Room 7E16, Ann Arbor, MI 48109 USA; 2 Medical Director, Scientific Therapeutics Information, Inc., 505 Morris Avenue – 3rd Floor, Springfield, NJ 07081 USA; 3 Senior Medical Director, Scientific Therapeutics Information, Inc., 505 Morris Avenue – 3rd Floor, Springfield, NJ 07081 USA

## Abstract

The risk of drug interactions with concurrent use of multiple medications is a clinically relevant issue. Many patients are unaware that over-the-counter (OTC) analgesics can cause potentially serious adverse effects when used in combination with other common medications such as anticoagulants, corticosteroids, or antihypertensive agents. Of particular significance is the increased risk of upper abdominal gastrointestinal adverse events in patients who take traditional nonsteroidal anti-inflammatory drugs (NSAIDs). This risk is dose dependent and further increased in patients who take more than one NSAID or use NSAIDs in combination with certain other medications. Some NSAIDs may also mitigate the antiplatelet benefits of aspirin and may increase blood pressure in patients with hypertension. Clinicians should be aware of potential drug interactions with OTC analgesics when prescribing new medications. Additionally, patients should be properly counseled on the appropriate and safe use of OTC analgesics.

## Introduction

A survey of medication use patterns in the United States has found that more than 80% of American adults used at least one over-the-counter (OTC) or prescription drug each week, and that 25% used at least 5 [1]. The OTC analgesics acetaminophen, ibuprofen, and aspirin are among the most frequently utilized medications, used by approximately 17% to 23% of the population each week. Chronic OTC analgesic use is most common in the elderly, many of whom take nonsteroidal anti-inflammatory drugs (NSAIDs) or acetaminophen for relief of pain. In addition, a recent survey reported that approximately 50% of American adults classified as having high cardiovascular (CV) risk status take low-dose aspirin for CV prophylaxis [1-3].

Because of the widespread availability and perceived safety of OTC analgesics, self-medication with these agents has become commonplace. Many patients are unaware of the potential for toxicity and adverse drug interactions associated with the long-term and inappropriate use of OTC analgesics. They may use OTC analgesics in higher-than-recommended doses or in combinations that magnify the risk of adverse interactions. Additionally, patients may not be aware that common cough, cold, or flu medications can contain OTC analgesics. Although OTC analgesics are associated with adverse effects in only a small percentage of people, the widespread use of these drugs makes even a small increase in population risk a clinically relevant issue [4]. Physicians can help patients avoid possible drug-drug interactions with commonly used OTC analgesics by providing counseling on the proper use of these agents.

## Currently available OTC oral analgesics and mechanisms of action

There are currently 4 OTC oral analgesics available in the United States: acetaminophen, aspirin, ibuprofen, and naproxen [5]. When taken as recommended, these OTC analgesics present relatively safe, effective, and economical treatments for mild to moderate pain, inflammation, and fever. Nevertheless, as a result of their accessibility and presumed safety, OTC analgesics are among the most commonly ingested drugs in overdoses [6].

Acetaminophen is generally considered to exert its analgesic effects through the inhibition of prostaglandin (PG) synthesis in the central nervous system [7], although the exact mechanism is not clearly defined. Several recent studies [7,8] have suggested alternative pathways, including peripheral elevation of the pain threshold. Aspirin and other NSAIDs inhibit the cyclooxygenase (COX) enzyme, thereby decreasing synthesis of PGs and related compounds that contribute to the inflammatory response and mediate a variety of cellular functions [9,10]. Traditional NSAIDs are nonselective for the 2 subtypes of the COX enzyme, although aspirin is 170-fold more potent in inhibiting COX-1 than COX-2 [9]. Whereas COX-1 inhibition by traditional NSAIDs is reversible, aspirin completely inactivates and irreversibly inhibits platelet COX-1, thus preventing formation of thromboxane A_2_[2,9].

## Potential drug interactions with OTC analgesics

Several potential drug-drug interactions should be considered when OTC analgesics are used in combination with other drugs (Table 1). In this article, these interactions are classified into 3 main groups: 1) increased gastrointestinal (GI) bleeding risk; 2) interference with the antiplatelet effects of aspirin; and 3) other potential interactions and issues.

**Table 1:** Potential drug interactions with OTC analgesics[5,42,43]

Drug combinations	Effect	Management options/considerations
Aspirin and NSAIDs or multiple NSAIDs	Increased risk of serious GI complications. Risk increases with increased dose and number of agents	Avoid concurrent use of more than one NSAID, if possible. Consider adding gastroprotective agents
Anticoagulants and NSAIDs	Increased risk of bleeding (especially GI) and increased oral warfarin activity	Avoid concurrent use of NSAID; monitor prothrombin time and occult blood in urine and stool
Corticosteroids and NSAIDs	Increased GI side effects, including ulceration and hemorrhage	Avoid concurrent use of NSAID and consider adding a gastroprotective agent
SSRIs and NSAIDs	Increased risk of GI bleeding	Avoid concurrent use of NSAID
Aspirin and ibuprofen or naproxen	Reduced antiplatelet effects of aspirin	Not seen with other NSAIDs or acetaminophen
Antihypertensive agents and NSAIDs	Use of NSAIDs may increase blood pressure	Monitor blood pressure and cardiac function
Antidiabetic agents (eg, sulfonylureas) and aspirin	Increased hypoglycemic effect	Avoid concurrent use and monitor blood glucose concentration
Lithium and NSAIDs	Increased steady-state lithium concentration and lithium toxicity	Monitor lithium concentrations. Interactions are less likely with aspirin than with naproxen or ibuprofen
Methotrexate and NSAIDs	Reduced renal clearance. Increased plasma methotrexate concentration	Avoid NSAIDs with high-dose methotrexate

GI = gastrointestinal; NSAID = nonsteroidal anti-inflammatory drug; OTC = over the counter; SSRI = selective serotonin reuptake inhibitor.

### Increased GI bleeding risk

Inhibition of COX by aspirin and other NSAIDs interferes with the production of protective mucosal PGs [9]. This mechanism likely explains the increased incidence of gastric ulcers and upper GI bleeding with use of NSAIDs. Listed below are several risk factors that increase the likelihood of developing GI toxicity with NSAIDs use [11-13]:

• Advanced age

• History of GI events

• Increased NSAID dose or multiple NSAID use

• Concomitant aspirin use.

Elderly patients are at greater risk of developing GI complications and often have comorbidities that require analgesic treatment [14]. Thus, careful monitoring of the amount of OTC and prescription NSAID consumption is imperative in the management of elderly patients. To minimize GI adverse events, proton-pump inhibitors (PPIs) or other gastroprotective agents may be useful for patients who require NSAIDs for anti-inflammatory therapy and are at risk for increased GI events [14,15].

**Figure 1:**
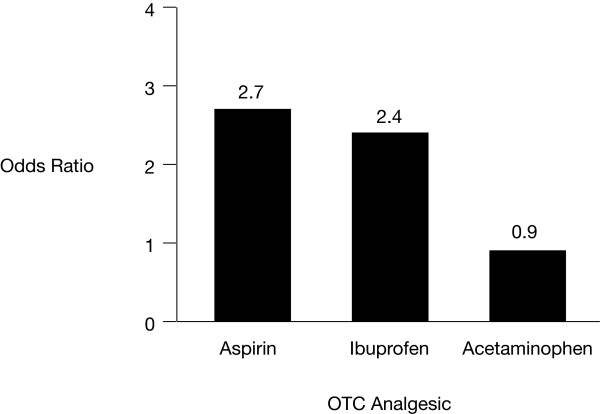
The risk for upper GI bleeding with aspirin, ibuprofen, and acetaminophen (adapted from reference [4], with permission). OTC = over the counter.

COX inhibition by aspirin results in a dramatic reduction of gastroprotective PGs in the GI tract, which explains the dose-dependent increase in GI adverse effects with higher doses of aspirin (odds ratio [OR] = 1.5 – 3.1) [16-18]. Several studies have reported that the use of enteric-coated and buffered low-dose aspirin does not appear to decrease the risk of GI adverse events (relative risk [RR], range = 2.3–2.7 {1.6–3.2}) [18-20]. Similarly, traditional NSAIDs are also associated with a 2- to 4-fold increased risk of GI side effects [11,16]. A summary of the risks for upper GI bleeding from a case-control study that included aspirin, acetaminophen, and ibuprofen, is shown in Figure 1[16]. Increased risk of GI adverse effects was observed with use of aspirin and ibuprofen, but not with acetaminophen.

Adverse GI effects are further compounded by the use of more than one agent and with higher NSAID doses [11,13,16]. Concomitant use of aspirin and traditional NSAIDs can double the risk of GI toxicity, as reported in a study of low-dose aspirin used for CV prophylaxis [18]. The standardized incidence rate of upper GI bleeding was 2.6 (95% confidence interval [CI]: 1.8–3.5) for patients using low-dose aspirin alone and 5.6 (95% CI: 4.4–7.0) for those using low-dose aspirin in addition to traditional NSAIDs. Gutthann and colleagues [11] reported much higher incidence of upper GI bleeding or perforation in patients who used multiple NSAIDs (adjusted OR = 9.0 [95% CI: 5.9–13.6]).

Although there is little inherent risk of GI events with anticoagulant use, several studies have shown that concomitant treatment with aspirin and other NSAIDs can increase the risk of GI hemorrhage and perforation [12,13]. This may be linked to the impairment of platelet aggregation induced by aspirin and nonselective NSAIDs [21]. A study by Shorr and colleagues [12] reported a nearly 13-fold increase (95% CI: 6.3–25.7) in risk of developing hemorrhagic peptic ulcer disease with concurrent use of NSAIDs and anticoagulants in patients aged 65 years and older. A similar study conducted in patients aged 25 to 80 years reported an adjusted relative risk (RR) of 6.4 (95% CI: 2.8–14.6) for developing upper GI bleeding and perforation in patients using an NSAID and an anticoagulant, compared with those who had not received either drug [13].

Corticosteroids may cause decreased gastric mucus production and delayed healing of NSAID-induced erosions [22]. The use of corticosteroids in patients not receiving NSAIDs is linked to a modest increase in GI events (OR range, 1.1 – 2.3) [11,22]. Nevertheless, there is wide reporting in the literature of a dramatic increase in the risk of adverse GI effects, including ulceration and hemorrhage, with concomitant current use of corticosteroids and NSAIDs (OR range, 2.2 – 14.6, in various studies) [11,13,22]. Piper and colleagues (1991) demonstrated that patients receiving corticosteroids (eg, cortisone 25 mg, hydrocortisone 20 mg, prednisolone 5 mg, prednisone 5 mg) in combination with NSAIDs had a 15 times greater risk for peptic ulcer disease than that of nonusers of either drug. Thus, although corticosteroids may not inherently increase the risk of GI toxicity, they exacerbate the risk posed by NSAIDs by delaying the healing of NSAID-induced ulcers.

Selective serotonin reuptake inhibitors (SSRIs) have been associated with an increased risk of upper GI bleeding [23,24]. Serotonin is essential in initiating the hemostatic response of platelets to vascular injury [25]. By blocking platelet uptake of serotonin, SSRIs may attenuate their function. Thus, SSRIs may impair hemostatic function and exacerbate underlying GI conditions when used concomitantly with drugs that cause GI ulceration and bleeding (eg, NSAIDs) [23]. A recent case-control study reported a low risk of GI adverse events with use of SSRIs (OR = 1.30 [95% CI: 1.13–1.50], compared with nonuse of SSRIs or NSAIDs), but confirmed an increased risk with concomitant use of NSAIDs (OR = 4.19 [95% CI: 3.30–5.31], compared with nonuse of either drug) [25].

Acetaminophen may be an effective alternative to NSAIDs for patients who require an analgesic and who are on concomitant aspirin, anticoagulant, corticosteroid, or SSRI therapy. Patients should be educated on the risk factors for developing adverse GI events with use of NSAIDs and on appropriate ways to minimize further risks.

### Interference with the antiplatelet effects of aspirin

**Figure 2:**
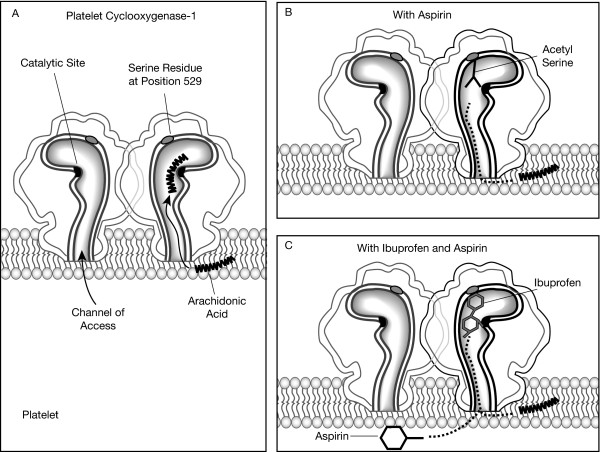
The effect of aspirin alone and of ibuprofen plus aspirin on platelet cyclooxygenase-1 (COX-1). A) The platelet prostaglandin (PG) G/H synthase-1 (COX-1) is depicted as a dimer, and the arachidonic acid substrate gains access to the catalytic site through a hydrophobic channel that leads to the core of the enzyme. B) Aspirin works by inhibiting access of arachidonic acid to the catalytic site by irreversibly acetylating a serine residue at position 529 in platelet COX-1. Interpolation of the bulky acetyl residue then permanently prevents metabolism of arachidonic acid into the cyclic endoperoxides PGG2 and PGH2. Because PGH2 is metabolized by thromboxane synthase into thromboxane A_2_, aspirin prevents the formation of thromboxane A_2 _by the platelets until new platelets are created. C) Nonsteroidal anti-inflammatory drugs (NSAIDs), such as ibuprofen, are competitive inhibitors of the catalytic site, and cause the reversible inhibition of thromboxane A_2 _formation during the dosing interval. Therefore, prior occupancy of the catalytic site by ibuprofen prevents aspirin from gaining access to its target serine (reproduced from reference [26], with permission).

Many patients use low-dose aspirin for primary or secondary prevention of myocardial infarction and stroke. Aspirin induces irreversible COX-1 inhibition in the platelet, a process that in turn inhibits the formation of thromboxane A_2 _and prevents platelet aggregation (Figure 2) [2,26]. Because aspirin completely inactivates platelet COX-1, antiplatelet benefits last for the lifetime of the platelet and are only attenuated by the regeneration of new platelets. As a result, even low doses of aspirin may provide beneficial CV effects. Nevertheless, recent studies have shown that traditional NSAIDs may interfere with the antiplatelet effects of aspirin by providing competition for the platelet COX-1 binding site [26-28]. This may limit the utility of aspirin as a cardioprotective agent in patients who require certain NSAIDs to manage pain effectively.

Numerous studies have shown that ibuprofen interferes with the antiplatelet effects of aspirin [26,28,29]. Catella-Lawson and colleagues [26] evaluated whether the antiplatelet effects of aspirin were mitigated by the concurrent use of ibuprofen, diclofenac, rofecoxib, and acetaminophen. Ibuprofen, administered as a single 400 mg dose given before aspirin (81 mg) or as 3 400 mg doses after a single 81 mg dose of aspirin, blocked the irreversible inhibition of platelet aggregation by aspirin. In contrast, the concomitant administration of rofecoxib (25 mg once daily), diclofenac (75 mg twice daily), or acetaminophen (1000 mg once daily) – administered either before or after a dose of aspirin – did not affect the inhibition of platelet aggregation by aspirin. Another study reported an increased risk in recurrent acute myocardial infarction with prolonged use of ibuprofen and aspirin compared with use of aspirin alone (hazard ratio for duration of exposure for at least 60 days = 1.83 [0.76–4.42]) [28]. Patients who take ibuprofen in addition to low-dose aspirin for CV prophylaxis should be mindful of the potential for drug interactions that may undermine the cardioprotective benefits of aspirin.

Recent studies on the effect of naproxen on platelet and vascular prostanoid inhibition have yielded inconclusive results [27,30]. Unlike aspirin, naproxen inhibits prostacyclin synthesis, the clinical implication of which is currently unknown. Nonetheless, naproxen is commonly suggested as an alternative to ibuprofen in patients with established CV risk who take daily low-dose aspirin for CV prophylaxis [15]. Other alternatives for analgesia for patients receiving long-term low-dose aspirin therapy include acetaminophen or other NSAIDs such as diclofenac, which do not preferentially inhibit COX-1. Because acetaminophen is a weak inhibitor of COX-1, it does not interfere with aspirin-mediated antiplatelet effects [7].

### Other potential interactions and issues

By inhibiting prostaglandin synthesis, NSAIDs can induce sodium retention and vasoconstriction [31]. Clinical studies have linked the use of NSAIDs to elevated blood pressure, particularly in patients with a history of hypertension who are already on antihypertensive medications [32-35]. In a meta-analysis of randomized trials studying the effect of NSAIDs on blood pressure, NSAIDs raised mean blood pressure by 5.0 mm Hg [34]. Patients who were concomitantly using β-blockers experienced greater elevations in mean blood pressure (6.2 mm Hg) compared with those using either vasodilators or diuretics. Careful monitoring of blood pressure and cardiac function is therefore recommended for hypertensive patients when initiating NSAID therapy. The potential risk of CV events with use of NSAIDs has been studied extensively in recent years. In 2005, the US Food and Drug Administration issued a request that manufacturers of all nonaspirin NSAIDs, including COX-2 inhibitors, revise package inserts to include a black box warning highlighting the increased risk for CV events and GI bleeding with use of these drugs [36].

Other medications may have adverse interactions when taken in conjunction with OTC analgesics (Table 1). Aspirin, particularly in combination with anticoagulation therapy, has been shown to increase the risk of intracerebral hemorrhages (ICH) [37]. Although ICH is an uncommon adverse effect of aspirin, the morbidity associated with this condition makes it a clinically relevant issue. Concomitant use of NSAIDs and antidiabetic agents, particularly sulfonylureas, may increase the risk of transient hypoglycemia [38]. NSAIDs have also been shown to increase risk of lithium and methotrexate toxicity by increasing drug concentrations to unsafe levels [39-41]. Monitoring drug concentrations and adjusting dosages when necessary may reduce the likelihood of adverse drug interactions with use of OTC analgesics.

## Approach to patient management

The following stepwise approach may be useful in the management of patients who routinely use OTC analgesics (Table 2) [15]. In a patient who has no established CV risk factors, with low or no NSAID GI risk, and who is not taking aspirin, nonselective NSAIDs may be used to manage pain or inflammation. For a patient who has no CV risk factors but who is at risk for NSAID-induced GI bleeding, a COX-2 selective inhibitor may be prescribed. Alternatively, the clinician may choose to prescribe a nonselective NSAID with a PPI or, in the case of a patient who has had prior GI bleeding, a COX-2 selective inhibitor with a PPI. Acetaminophen may be recommended for patients at risk for GI events or for those who have a history of adverse effects with aspirin or NSAID use.

**Table 2:** Clinicians' guide to anti-inflammatory therapy (reproduced from reference [15], with permission)

	No or low NSAID gastrointestinal risk	NSAID gastrointestinal risk
No cardiovascular risk (without aspirin)	Nonselective NSAID (cost consideration)	COX-2 selective inhibitor or nonselective NSAID and proton-pump inhibitor
		COX-2 selective inhibitor and proton-pump inhibitor for those with prior GI bleeding
Cardiovascular risk (with aspirin)	Naproxen^a^	Proton-pump inhibitor irrespective of NSAID
	Addition of proton-pump inhibitor if gastrointestinal risk of aspirin/NSAID combination warrants gastroprotection	Naproxen if CV risk outweighs GI risk
		COX-2 selective inhibitor and proton-pump inhibitor for those with previous GI bleeding

COX = cyclooxygenase; CV = cardiovascular; GI = gastrointestinal; NSAID = nonsteroidal anti-inflammatory drug.

^a^Nonselective or selective (low-dose) inhibitor without established aspirin interaction if naproxen is ineffective.

Misoprostol at full dose (200 μg four times a day) may be substituted for proton-pump inhibitor.

A patient with established CV risk factors (taking aspirin for CV prophylaxis) who is at minimal risk of NSAID-induced GI complications may use naproxen or another NSAID without established aspirin interaction. A PPI may be added to this regimen should the combination of aspirin and NSAID warrant gastroprotection. Patients with established CV and GI risk factors should receive a PPI to be used in conjunction with NSAIDs. If the CV risk factors outweigh the GI risk factors, naproxen is recommended. As above, in a patient who has had previous GI bleeding, the clinician should suggest the use of a COX-2 selective inhibitor.

Additional recommendations include monitoring blood pressure and cardiac function in patients with hypertension. In diabetic patients receiving sulfonylureas and NSAIDs (including aspirin), routine checks for signals of increased hypoglycemia should be performed. All patients should be educated on potential drug interactions that may occur with OTC analgesics and prescription medications.

## Conclusion

Because many patients self-medicate with OTC analgesics and are unaware of potentially dangerous drug interactions, proper counseling on the appropriate use of these agents can help minimize adverse effects and ensure positive clinical outcomes.

## Competing interests

A. Mark Fendrick has no competing interests to disclose.

Deborah E. Pan and Grace E. Johnson are employees of Scientific Therapeutics Information (STI), Springfield, New Jersey. STI received funding from McNeil Consumer Healthcare to provide writing or editorial assistance on this manuscript.

## Authors' contributions

AMF, DEP, and GEJ were involved in the conception, drafting, revising, and final approval of the important intellectual content for this manuscript.
